# Synthesis of New Polyfluoro Oligonucleotides via Staudinger Reaction

**DOI:** 10.3390/ijms26010300

**Published:** 2024-12-31

**Authors:** Kristina Klabenkova, Alyona Zakhryamina, Ekaterina Burakova, Sergei Bizyaev, Alesya Fokina, Dmitry Stetsenko

**Affiliations:** 1 Department of Physics, Novosibirsk State University, 2 Pirogov Str., Novosibirsk 630090, Russia; k.klabenkova@g.nsu.ru (K.K.); burakovaea@bionet.nsc.ru (E.B.); bizyaevsn@bionet.nsc.ru (S.B.); fokinaaa@bionet.nsc.ru (A.F.); 2 Institute of Cytology and Genetics, Siberian Branch of the Russian Academy of Sciences, 10 Lavrentiev Ave., Novosibirsk 630090, Russia; 3 Department of Natural Sciences, Novosibirsk State University, 2 Pirogov Str., Novosibirsk 630090, Russia; zakhryamina95@mail.ru

**Keywords:** perfluoroalkane, nucleic acid, DNA, RNA, zwitter-ionic oligonucleotide, sulfonyl azide, cellular uptake

## Abstract

Nowadays, nucleic acid derivatives capable of modulating gene expression at the RNA level have gained widespread recognition as promising therapeutic agents. A suitable degree of biological stability of oligonucleotide therapeutics is required for in vivo application; this can be most expeditiously achieved by the chemical modification of the internucleotidic phosphate group, which may also affect their cellular uptake, tissue distribution and pharmacokinetics. Our group has previously developed a strategy for the chemical modification of the phosphate group via the Staudinger reaction on a solid phase of the intermediate dinucleoside phosphite triester and a range of, preferably, electron deficient organic azides such as sulfonyl azides during automated solid-phase DNA synthesis according to the conventional β-cyanoethyl phosphoramidite scheme. Polyfluoro compounds are characterized by unique properties that have prompted their extensive application both in industry and in scientific research. We report herein the synthesis and isolation of novel oligodeoxyribonucleotides incorporating internucleotidic perfluoro-1-octanesulfonyl phosphoramidate or 2,2,2-trifluoroethanesulfonyl phosphoramidate groups. In addition, novel oligonucleotide derivatives with fluorinated zwitterionic phosphate mimics were synthesized by a tandem methodology, which involved (*a*) the introduction of a carboxylic ester group at the internucleotidic position via the Staudinger reaction with methyl 2,2-difluoro-3-azidosulfonylacetate; and (*b*) treatment with an aliphatic diamine, e.g., 1,1-dimethylethylenediamine or 1,3-diaminopropane. It was further shown that the polyfluoro oligonucleotides obtained were able to form complementary duplexes with either DNA or RNA, which were not significantly differing in stability from the natural counterparts. Long-chain perfluoroalkyl oligonucleotides were taken up into cultured human cells in the absence of a transfection agent. It may be concluded that the polyfluoro oligonucleotides described here can represent a useful platform for designing oligonucleotide therapeutics.

## 1. Introduction

To date, there is no doubt that oligonucleotide therapeutics have a huge potential to account for unmet needs in the medical treatment of human diseases [1,2]. Oligonucleotides, which can selectively target cellular nucleic acids, typically, messenger or noncoding RNAs, through the complementary Watson–Crick bonding, exert a therapeutic influence at the pre- or, more often, post-transcriptional level [3,4]. Since 1998 and up to the end of 2023, the FDA has approved over twenty nucleic acid drugs, nearly three-quarters of them within the last seven years [5]. A majority of these act as antisense agents [6,7], e.g., nusinersen (Spinraza) [8], eteplirsen (Exondys 51) [9], etc. [10,11], or through the RNA interference (RNAi) mechanism [12,13] such as a small interfering RNA (siRNA) drug patisiran (Onpattro) [14] and others that followed [15,16,17,18]. The necessary stabilization of oligonucleotide therapeutics in biological media for the in vivo application is usually achieved by the chemical modification of various parts of the molecule: heterocyclic bases, furanose ring to give, e.g., 2′-*O*-methyl RNA [19,20], 2′-α- or β-fluoro-DNA [21,22], bridged/locked nucleic acids (B/LNAs) [23,24,25], etc. [26,27,28], or, in particular, phosphate backbone. The most well-studied and popular example of the latter is the phosphorothioate group (PS), which is a common feature of many antisense drugs [29], joined recently by mesyl phosphoramidate [30,31,32], by phosphoryl guanidine [33,34,35] and, previously, by a number of other phosphate mimics [36,37,38,39,40]. Some nucleic acid analogues represent further departures from the natural DNA structure such as peptide nucleic acids (PNAs) [41] or phosphordiamidate morpholino oligomers (PMOs) [42,43], the latter accounted for several drugs in the market [9,10,11].

However, one of the main challenges on the way of oligonucleotide therapeutics into clinical practice is the development of safe and efficient delivery systems [44,45,46,47,48,49]. To improve the efficacy of the intracellular transport of nucleic acids, many specially developed delivery vectors have been proposed based on either non-covalent, primarily, electrostatic, association with cationic lipids and polymers, in particular, polyethyleneimine (PEI) [50] and cationic dendrimers [51], or the formation of covalently linked conjugates with various transporter molecules such as lipophilic compounds [52,53,54], cell-penetrating peptides (CPPs) [55] and polyamines [56]. However, despite the obvious progress in this area [57,58], the development of a universal and inexpensive method for the effective delivery of oligonucleotide drugs to target organs or tissues, apart from the liver for GalNAc conjugates [59], is still an urgent and difficult task [60].

Fluorine atoms are essential constituents of many therapeutic drugs, and polyfluoro compounds are known to possess unique features, which made them indispensable in various fields of pharmaceutical, diagnostic and biomedical research [61]. Being chemically inert and amphiphobic, i.e., both hydrophobic and lipophobic, polyfluoro compounds tend to self-assemble into supramolecular structures due to fluorine–fluorine interactions [62]. This property was extensively exploited for drug delivery applications, especially for anticancer drugs [63], cytosolic protein delivery [64,65], transfection [66], CRISPR/Cas9 [67], mRNA vaccines [68] and, most recently, pH-responsive drug release coupled with ^19^F MRI detection [69], a decrease in immune response [70] and an increase in serum stability [71]. Previously, the polyfluorination of cationic PAMAM or PPI dendrimers was shown to result in the improved cellular uptake of complexed nucleic acids with only a marginal or no increase in toxicity [72,73,74,75]. Beneficial for low-toxic DNA and RNA delivery was also the polyfluorination of PEI [76,77,78,79] and other polymers [80]. Polyfluorinated cationic polymers were also shown to possess antibacterial activity against multidrug-resistant Gram-negative ESKAPE pathogens [81]. Furthermore, Metelev et al. showed that the direct incorporation of perfluorooctyl groups into oligonucleotides increased the melting temperature of their duplexes and improved cellular uptake in vitro [82], probably due to the strong association of polyfluoro fragments and the temporary destabilization of sections of the cytoplasmic membrane. Polyfluorinated tags were introduced into the oligonucleotides on the 5′-end using either 3-(1-perfluorooctyl)-propyl phosphoramidite during automated solid-phase synthesis or via post-synthetic conjugation of 3-(1-perfluorooctyl)-propylamine to an activated 5′-carboxy oligonucleotide on solid phase [82]. However, despite some promising results achieved in this work, the possibilities for the polyfluorination of oligonucleotides by using such a phosphoramidite as in Ref. [82] are limited to the terminal modifications in addition to being usually expensive and time-consuming due to the procurement of a non-standard monomer. Otherwise, it is necessary to use post-synthetic conjugation as an extra step, again limiting the placement of the modification to only the terminal positions of the oligonucleotide chain. We also speculated that polyfluoro oligonucleotides may show increased cellular uptake by spontaneously forming nanoparticles in aqueous solutions due to the self-association of polyfluorinated tags similar to hydrophobic tricyclic DNAs [83] and lipid–oligonucleotide conjugates [84].

The Staudinger reaction of internucleotidic phosphite triester and electrophilic organic azides is a facile way to a wide range of nucleic acid analogues with the P–N bond as was demonstrated by us [33,85,86,87] and then picked up by others [32,35,88,89,90]. It can potentially serve as a convenient alternative for the incorporation of perfluoroalkyl moieties at any internal position within the oligonucleotide chain. The reaction has been applied for the modification of nucleic acids as early as in 1975 [91]. In the mid-2000s, a pioneering work by Heindl et al. unfolded a convenient sulfonyl phosphoramidate chemistry [92,93], which was later successfully applied by us to obtain novel oligonucleotides with internucleotidic sulfonyl phosphoramidate groups such as mesyl phosphoramidates [87], which proved to be useful as antisense agents [30,31,32]. Later, a successful introduction of various reporter groups at selected internucleotidic positions via sulfonyl phosphoramidate chemistry was also reported [90].

In this communication, we describe the application of the Staudinger reaction and sulfonyl phosphoramidate chemistry to introduce one or more polyfluoroalkyl moieties into internucleotidic positions of an oligonucleotide chain. This approach afforded new derivatives, which can be expected to be taken up by cells in the absence of a transfection agent not only due to the unique *fluorous effect* [61,62] but also by reducing the total negative charge of the oligonucleotide via the introduction of zwitter-ionic groups [88] similarly to the recently described tandem Staudinger reaction and amide bond formation for the conjugation of oligonucleotides with amines [94], which was applied in this work for the preparation of fluorinated zwitter-ionic oligonucleotides. Notably, the observed nuclease resistance [30] and duration of the action [30,32] of the mesyl phosphoramidate oligonucleotides is expected to be retained, or even further augmented, in the polyfluoro analogues.

## 2. Results and Discussion

### 2.1. Synthesis and Characterization of New Polyfluorinated Oligonucleotide Derivatives

**Figure 1:**
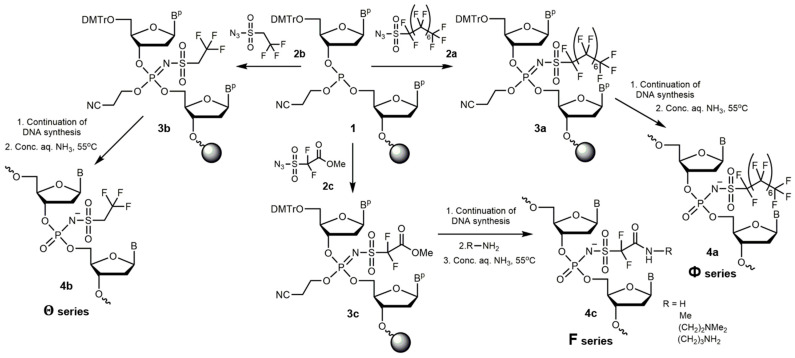
A general scheme for the preparation of polyfluoro oligonucleotides and their amide-linked conjugates (in the case of azide **2c**). Key: B^p^/B—protected/unprotected nucleobase (A, C, G or T); DMTr—4,4′-dimethoxytrityl; for examples of R, see Table 2.

The respective perfluoro-1-octanesulfonyl azide (**2a**), 2,2,2-trifluoroethanesulfonyl azide (**2b**) and methyl 2,2-difluoro-3-azidosulfonylacetate (**2c**) were easily obtained from corresponding sulfonyl fluorides (**2a** and **2c**) or a sulfonyl chloride (**2b**) by reaction with sodium azide. They were used for the introduction of polyfluoroalkyl groups into oligonucleotides via the Staudinger reaction. Notably, azide **2c** carries a reactive carboxylic ester group, which after treatment with a diamine provided access to zwitter-ionic polyfluoro oligonucleotides. The modification of oligonucleotides can be carried out at any internucleotidic position during automatic synthesis by replacing the standard oxidation with aqueous iodine by the Staudinger reaction with the solution of azide **2a**–**c** placed into a synthesizer bottle (Figure 1). The reactions were not optimized; all three azides were coupled under a similar protocol, leading in the case of perfluorooctyl azide **2a** to moderate yields after 30 min. However, azides **2b** and **2c** resulted in higher yields of crude oligonucleotides, which allowed us to proceed up to the full substitution at every internucleotidic position with either group, with reasonably good prospects for the scalability and cost-effectiveness of the synthesis at a larger scale. Typically, oligonucleotides were synthesized with the retention of 5′-dimethoxytrityl group (“DMTr On” mode), and the respective 5′-DMTr-containing fraction was isolated using RP-HPLC (with the exception of azide **2c**). The DMTr group was subsequently cleaved by treatment with 80% acetic acid at an ambient temperature for 15 min, or for the oligonucleotides containing perfluorooctyl residues, by treatment with 10 mM HCl at ambient temperature for 15 min. The structures of modified oligonucleotides were confirmed using MALDI-TOF MS (Supplementary File, Table S1).

Oligodeoxy- and oligo-2′-*O*-methylribonucleotides with one or two 2,2,2-trifluoroethyl groups and the sequences with every internucleotidic position modified were obtained from azide **2b** (Table 1). Oligodeoxynucleotides containing one or two perfluorooctyl groups were obtained from azide **2a** (Table 1), including 17 nt sequences with a 6-carboxamido fluorescein (Flu) residue at the 3′- or 5′- end for cellular uptake studies using laser confocal fluorescent microscopy. Final deprotection and cleavage from the support was carried out with conc. aqueous ammonia at 25 °C for 1 h for hexathymidylates or at 55 °C for 12 h for all other oligonucleotides.

**Figure 2:**
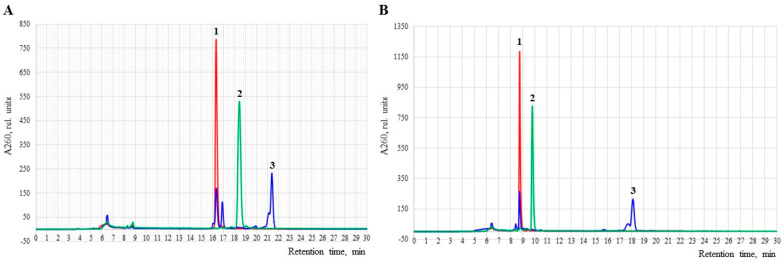
RP-HPLC elution profiles of crude oligonucleotides: (**A**) unmodified oligonucleotide 5′−DMTr−dT_6_ (**1**), **Θ1** (**2**) and **Φ1** (**3**); (**B**) unmodified oligonucleotide 5′−d(TTTTTT) (**1**), **Θ2** (**2**) and **Φ2** (**3**). Elution gradient I (Section 4).

Typical RP-HPLC elution profiles of reaction mixtures of model hexathymidylates containing 2,2,2-trifluoroethyl (**Θ1**, **Θ2**) or perfluorooctyl (**Φ1**, **Φ2**) groups on the 5′-end are shown in Figure 2. The appearance of an internucleotidic trifluoroethyl group, as expected, led to an increase in retention time compared to the unmodified control (Figure 2, 2 vs. 1). For longer perfluorooctyl groups, the retention time has increased even more to allow clean separation from unmodified sequences (Figure 2, 3 vs. 1). The separation of the main product peak into diastereomers was detected only in the case of perfluorooctyl oligonucleotides **Φ1** and **Φ2** in which, in addition, byproduct peaks were present due to an incomplete Staudinger reaction.

Support-bound oligonucleotides with methyl difluoroacetate group (**3c**) at various internucleotidic positions were produced with azide **2c** during automated synthesis. Following the aminolysis of the ester group, a number of amide-linked fluorinated oligonucleotides (**4c**) were obtained (Table 2). To prevent the premature hydrolysis of the ester, some of the samples were treated with a 2M solution of ammonia in 2-propanol (***i***) for 18 h at 55 °C affording oligonucleotides **F1** with the 5′-DMTr group and **F2** without, or with ethanolic methylamine (***ii***) for 1 h at ambient temperature, producing 5′-DMTr-containing **F3** and its DMTr Off counterpart **F4**. By treating the samples with 1,1-dimethylethylenediamine (***iii***) or 1,3-diaminopropane (***iv***) for 1 h at 55 °C, we obtained oligonucleotides **F5**–**F15** and **F16**–**F21** with zwitter-ionic groups containing tertiary or primary amino groups in the side chains, respectively, including some sequences with zwitter-ionic groups at every internucleotidic position (**F10**, **F21**). In these two, the side-chain amino groups, which are expected to be protonated within physiological pH range, compensated the negative charge of the sulfonyl phosphoramidate group, thereby reducing the net charge of the molecule to zero as compared to the fully anionic character of the unmodified oligonucleotide.

**Figure 3:**
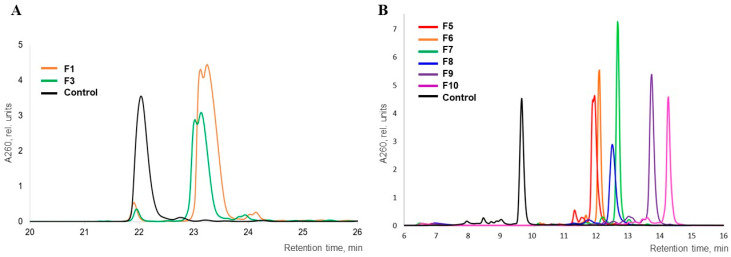
RP-HPLC elution profiles: (**A**) DMTr-conjugates **F1** and **F3** treated with 2M NH_3_/Pr*^i^*OH or CH_3_NH_2_/EtOH, respectively, compared to unmodified oligonucleotide 5′-DMTr-dT_6_ (control); elution gradient (I); (**B**) conjugates **F5**–**F10** with 1,1-dimethylethylenediamine, compared to unmodified oligonucleotide dT_6_ (control); elution gradient (II).

An increase in the RP-HPLC retention time was registered for oligonucleotides **F1** and **F3** compared to the unmodified control (Figure 3A) as well as for those without the DMTr group (**F2** and **F4**) (Supporting Material, Figure S1A). The separation of diastereomers was observed in all the samples retaining the DMTr group but only for a 5′-singly modified hexathymidylate **F5** out of the DMT Off sequences. The difference between the retention times of the pairs of samples **F1/F3** (Figure 3A) and **F2/F4** (Figure S1A) differing in a single methyl group was insignificant. With the increase in the number of zwitter-ionic groups in the molecule and the concomitant decrease in the total negative charge, we observed a gradual rise in the RP-HPLC retention time of the hexathymidylates with 1,1-dimethylethylenediamine side chains **F5**–**F10** (Figure 3B), as well as those with 1,3-diaminopropane **F16**–**F21** side chains (Supporting Material, Figure S1B). The treatment time of only 1 h was deemed to be insufficient for the complete incorporation of four and five 3-aminopropyl groups into oligonucleotides **F20** and **F21**, which resulted in the appearance of side products in the RP-HPLC profile. Nevertheless, the corresponding 15 nt oligonucleotides with one or two *N*,*N*-dimethylaminoethyl groups **F11**–**F15** were isolated in high yields (Supporting Material, Figure S2) and were used to study the thermal stability of complementary duplexes with DNA and RNA (see Section 2.3).

### 2.2. Electrophoretic Mobility of the Polyfluoro Oligonucleotides

**Figure 4:**
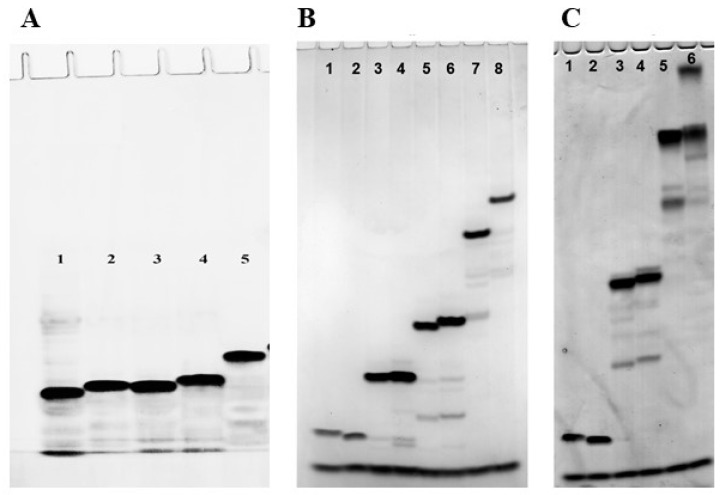
Electrophoretic mobility comparison: (**A**) of the oligonucleotides with 2,2,2-trifluoroethanesulfonyl phosphoramidate groups, lanes: (1) unmodified 5′-d(GCGCCAAACA), (2) **Θ3**, (3) **Θ4**, (4) **Θ5**, (5) **Θ6**; (**B**) of the conjugates with 1,1-dimethylethylenediamine, lanes: (1) dT_5_, (2) dT_6_, (3) **F5**, (4) **F6**, (5) **F7**, (6) **F8**, (7) **F9**, (8) **F10**; (**C**) of the conjugates with 1,3-diaminopropane, lanes: (1) dT_5_, (2) dT_6_, (3) **F18**, (4) **F19**, (5) **F20**, (6) **F21**.

The mobility of the polyfluoro oligonucleotides was studied by 20% denaturing polyacrylamide gel electrophoresis (PAGE). A sample of 10-mer oligodeoxyribonucleotides, 5′-d(GCGCCAAACA), both unmodified and modified with one (**Θ3** and **Θ4**) or two (**Θ5**) 2,2,2-trifluoroethanesulfonyl phosphoramidate groups, as well as the fully modified sequence with a total of nine such groups (**Θ6**), was studied. The mobility of the modified oligonucleotides was close to the unmodified control apart from the fully modified sample **Θ6** with significantly higher molecular mass (Figure 4A), which indicated that the 2,2,2-trifluoroethanesulfonyl phosphoramide group in oligonucleotides, like previously studied sulfonyl phosphoramidate groups, retains a negative charge at physiological pH similar to the natural phosphodiester group [65,66].

Next, we assessed the difference in the PAGE mobility of oligonucleotides depending on the number of fluorinated zwitter-ionic groups (from 1 to 5). Reaction mixtures of the oligonucleotides **F5**–**F10** with *N*,*N*-dimethylaminoethyl groups (Figure 4B) and **F16**–**F21** with 3-aminopropyl groups (Figure 4C and Figures S3, lanes 7 and 8) were applied to the gel lanes in order of the increase in the number of zwitter-ionic groups next to the lanes of the unmodified 5′-dT_5_ and 5′-dT_6_ controls. The experimental results showed, as expected, a gradual decrease in electrophoretic mobility with the increase in the number of zwitter-ionic groups in the sequence, which correlates with a gradual decrease in the net-negative charge of the oligonucleotides. The retardation effect was more pronounced for the oligonucleotides with 3-aminopropyl residues. Yet, for the singly modified conjugates with the same amine, no visible difference in electrophoretic mobility was observed depending on the position of modification: either 3′- (**F5** or **F16**) or 5′-terminal (**F6** or **F17**) (Figure 4B, lanes 3 and 4, and Figure S3, lanes 7 and 8). For the oligonucleotides with two zwitter-ionic groups **F7/F8** or **F18/F19**, the location of the modifications at the opposite ends of the sequence led to a slight decrease in mobility compared to the location side by side at the 3′-end (Figure 4B and Figure 4C, lines 5–6 and 3–4, respectively). The exhaustive substitution of all of the phosphate groups with zwitter-ionic groups dramatically reduced the mobility (Figure 4B and Figure 4C, lanes 7–8 and 5–6, respectively) but, nevertheless, as in our previous work [73], did not result in the complete compensation of the net-negative charge of the molecule under the experimental conditions (pH 7.5), which suggests that some of the aminoalkyl side chains may have remained unprotonated.

### 2.3. Cytotoxicity of Polyfluoro Oligonucleotides to Human Breast Cancer Cells

**Figure 5:**
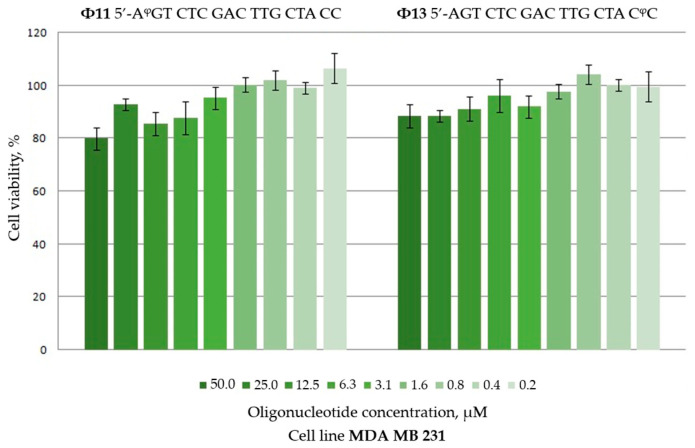
Viability of MDA MB 231 breast cancer cells at different concentrations of perfluorooctyl oligonucleotides **Φ11** and **Φ13** (see Table 1) according to MTT test.

The nonspecific cytotoxicity of oligonucleotides **Φ11** and **Φ13** containing one perfluoro-1-octanesulfonyl phosphoramidate group at either 5′- or 3′-end, respectively, for human breast cancer cells MDA MB 231 was assessed via the MTT test. Based on the results of the experiment, presented as the percentage of living cells compared to control cells (Figure 5), it can be concluded that the introduction of one perfluorooctyl group at either end does not result in a significant dose-dependent toxicity for the cancer cell line (unless at the highest concentration of 50 μM).

### 2.4. Thermal Denaturation Studies of Duplexes of Polyfluorinated Oligonucleotides with DNA and RNA

Prior to studying the influence of 2,2,2-trifluoroethanesulfonyl- and perfluoro-1-octanesulfonyl phosphoramidate groups as well as their zwitter-ionic counterparts on the thermal stability of complementary duplexes with DNA and RNA, the corresponding oligonucleotides were analyzed by RP-HPLC and purified by PAGE under denaturing conditions. When a single trifluoroethyl group was introduced into an oligonucleotide, there was no adverse effect on the thermal stability of its duplexes with either DNA or RNA compared to unmodified control (Table 3, blue background). However, the exhaustive replacement of all of the phosphate groups with 2,2,2-trifluoroethanesulfonyl phosphoramidate groups led to a more pronounced destabilization of a duplex with RNA (**Θ6**) and where both complementary strands were fully substituted with trifluoroethyl groups (**Θ6**:**Θ7**). One or two fluorinated *N*,*N*-dimetylaminoethyl zwitter-ionic groups in the oligonucleotides also had a very slight effect on the formation of duplexes with RNA (Table 3, gray background).

The introduction of one perfluoro-1-octanesulfonyl phosphoramidate modification either at the 5′-end or in the middle also had no effect on the thermal stability of duplexes formed with DNA by a 10 nt oligonucleotides (**Φ3**, **Φ4**). However, the formation of blunt dT_20_:dA_20_ DNA duplexes led to some destabilizing effect (**Φ9**), more pronounced when there were perfluooctyls in either strand opposite each other (**Φ9**:**Φ10**) (Table 3, green background). In the 10 nt DNA duplex, the destabilization was greater when two perfluorooctyl groups were located in either strand directly opposite each other (**Φ4:Φ7**) and less when one of the prefluorooctyls was shifted to the next base pair (**Φ3:Φ6**). Furthermore, when two perfluoro-1-octanesulfonyl phosphoramidate groups were introduced into the same oligonucleotide dT_20_, no DNA duplex formation with dA_20_ was detected. Thus, our initial hypothesis that the location of polyfluorocarbon residues opposite each other may have a stabilizing effect on the formation of a complementary duplex, as was the case with hydrocarbon chains, was not justified for the relatively short perfluorooctyl group. Nevertheless, the data obtained indicate that the oligonucleotides modified with fluorinated internucleotidic groups retain the ability to form stable complementary duplexes with DNA and RNA although they were slightly destabilized relative to the natural DNA–DNA and DNA–RNA duplexes. Notably, the zwitter-ionic fluorinated *N*,*N*-dimethylaminoethyl modifications were better tolerated in a duplex with RNA, which may have implications for targeting biological RNAs.

However, the fluorine substitution of the sulfonyl group, with the possible exception of a bulky perfluorooctyl residue, did not appear to result in a significant additional decrease in the thermal stability of the duplexes of the sulfonyl phosphoramidate oligonucleotides with either DNA or RNA as compared to other sulfonyl phosphoramidate modifications [86,87,88,90,94].

### 2.5. Laser Confocal Microscopy Study of the Uptake of Polyfluorinated Oligonucleotides into Human Breast Cancer Cells

**Figure 6:**
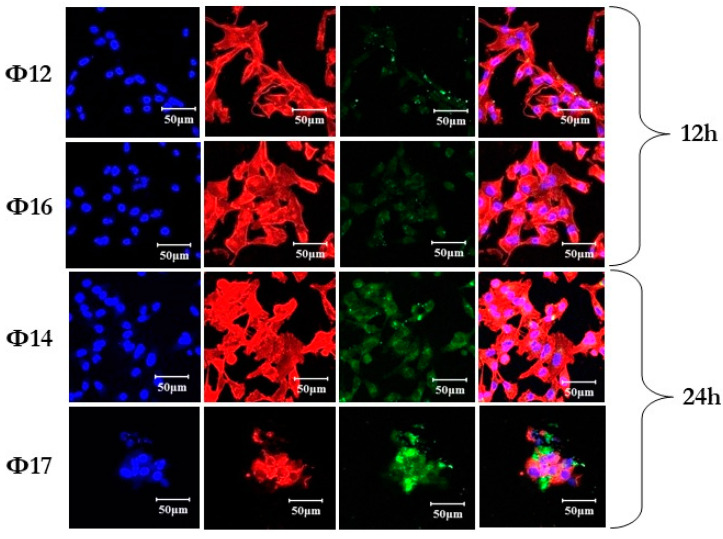
Laser confocal fluorescence microscopy study of the uptake of fluorescein-labeled oligonucleotides (5 μM) with perfluoro-1-octanesulfonyl phosphoramidate groups into MDA MB 231 human breast cancer cells. Oligonucleotides **Φ12**—5′-d(A**^φ^**GTCTCGACTTGCTACC-Flu), **Φ16**—5′-d(A**^φ^**GTCTCGACTTGCTAC**^φ^**C-Flu), **Φ14**—5′-d(Flu-AGTCTCGACTTGCTAC**^φ^**C) and **Φ17**—5′-d(Flu-A**^φ^**GTCTCGACTTGCTAC**^φ^**C). Blue channel: cell nuclei stained with DAPI; red channel: actin filaments stained with phalloidin-TRITC; green channel: oligonucleotides labeled with 6-carboxamidofluorescein; the rightmost column: an overlap of all the channels.

Next, we studied the ability of fluorescein-labeled 17 nt oligonucleotides containing one or two perfluoro-1-octanesulfonyl phosphoramidate groups to be taken up into human cells in the absence of a transfection agent. For this, MDA MB 231 cells were incubated with modified oligonucleotides at a concentration of 5.0 μM for 12 h (**Φ12**, **Φ16**) or 24 h (**Φ14**, **Φ17**). It was shown that oligonucleotides modified with perfluorooctyl groups are capable of slowly penetrating cells in the absence of a transfection agent and gradually accumulating inside as the incubation time increases from 12 to 24 h (Figure 6). The comparison of cellular uptake efficiency showed that the samples with one perfluorooctyl group are internalized predominantly into the cytoplasm with approximately the same efficiency regardless of the location of the modification at the 3′- or 5′-end. Meanwhile, the introduction of two perfluorooctyl groups mainly leads to the concentration of the oligonucleotide around the cell membrane, probably due to the ability of perfluorocarbon chains to self-associate, which led to slower cellular uptake. It will be interesting to compare the cell-penetrating ability of fluorocarbon chains vs. hydrocarbon chains (as per Ref. [84]), and we see this as a future avenue of our studies.

## 3. Materials and Methods

### 3.1. Synthesis of Perfluoro-1-Octanesulfonyl Azide, 2,2,2-Trifluoroethanesulfonyl Azide and 2,2-Difluoro-3-Azidosulfonylacetate

Perfluoro-1-octanesulfonyl azide (**2a**) 2,2,2-trifluoroethanesulfonyl (tresyl) azide (**2b**) and methyl 2,2-difluoro-3-azidosulfonylacetate (**2c**) were obtained from perfluoro-1-octanesulfonyl fluoride, tresyl chloride (both Sigma-Aldrich Inc., St Louis, MO, USA) and methyl 2,2-difluoro-2-(fluorosulfonyl)-acetate (Fluorochem, Hadfield, UK) by reaction with NaN_3_ (SoyuzKhimProm, Barnaul, Russia) in dry acetonitrile (UHPLC Supergradient, PanReac, Madrid, Spain) (see Supplementary Material for general information, experimental details, and NMR spectra (Figures S4–S12)).

### 3.2. Synthesis of Oligonucleotides

Oligonucleotides were synthesized on an automated DNA/RNA ASM-800 synthesizer (Biosset Ltd., Novosibirsk, Russia) according to the phosphoramidite method at 0.2 or 0.4 μM scale using the corresponding deoxyribonucleotide 2-cyanoethyl *N*,*N*-diisopropyl phosphoramidites with standard protection: benzoyl for dA and dC, isobutyryl for dG (Sigma-Aldrich Inc., St Louis, MO, USA) and Controlled Pore Glass (CPG) 500 Å supports with attached protected deoxyribonucleosides (Glen Research Corp, Sterling, VA, USA). For the incorporation of the modification during automated synthesis, a solution of the corresponding azide in dry acetonitrile (0.25 M) was placed into a synthesizer bottle and pumped to the column instead of iodine oxidizer solution for the period of 30 min. After the synthesis, the support-bound oligonucleotides were treated with conc. aq. ammonia solution at 25 °C for 1 h for hexathymidylates, or at 55 °C for 12 h for all other oligonucleotides, or passed on to the conjugation step (see Section 3.3).

If oligonucleotides were synthesized with 5′-dimethoxytrityl group retained (“DMTr On” mode), the 5′-DMTr-containing fraction was isolated using RP-HPLC (see Section 3.4). The DMTr group was then cleaved by treatment with 80% acetic acid at ambient temperature for 15 min, or for oligonucleotides containing perfluoro-1-octyl residues, by treatment with 10 mM HCl at ambient temperature for 15 min.

### 3.3. Synthesis of Oligonucleotide Conjugates

For conjugation reaction, a 60 µL of a solution of the desired amine **1**–**4** was added to the support-bound oligonucleotides. The mixture was shaken for 18 h at 55 °C for amines **1**–**2** and for amines **3**–**4**. 1,1-Dimethylethylenediamine (**3**) and 1,3-diaminopropane (**4**) were used neat (100 μL per column) to carry out the conjugation, final deprotection and cleavage from solid support in a single step. After completion of the reaction, the volatiles were removed in vacuo. For the 15 nt oligonucleotide conjugates **F11**–**F15**, additional treatment with AMA solution (conc. aq. (~28%) ammonia−conc. aq. (~40%) methylamine, 1:1 *v*/*v*) at 55 °C for 10 min was carried out to ensure the complete removal of the isobutyryl protecting group from dG residues. Then, the conjugates were dissolved in 100 μL of 20 mM TEAA, pH 7.0 (100 L), carefully transferred into a new test tube, precipitated with lithium perchlorate solution (3% *w*/*v*) in acetone (1 mL) and kept at −20 °C for 30 min. After centrifugation (13,400 rpm, 4 min), the supernatant was discarded, and the pellet was washed with acetone (3 × 500 μL) and left to air-dry for 10 min. Finally, the conjugate was re-dissolved in 20 mM TEAA, pH 7.0 (100 μL), and characterized by RP-HPLC.

### 3.4. Reverse-Phase HPLC Analysis of Oligonucleotides and Conjugates

Analytical RP-HPLC was performed on Agilent 1220 (Agilent Technologies, Santa Clara, CF, USA) with the use of Zorbax Eclipse XDB-C18 column (4.6 × 150 mm, 5.5 µm bead size) for oligonucleotide series **Θ** and **Φ**; or on a Milichrom A02 system (Econova Ltd., Chromatography Institute, Novosibirsk, Russia) on a ProntoSil 120-5 C18 AQ column (2 × 75 mm, 5 µm) for oligonucleotide series **F**. The elution was carried out at a flow rate of 2 mL/min (for Agilent 1220) or 100 µL/min (for Milichrom A02) and UV detection at 260 and 280 nm. Two elution gradients of acetonitrile (UHPLC Supergradient, PanReac, Madrid, Spain) in 20 mM TEAA, pH 7.0, were applied: 0–50% in 30 min (I) and 0–60% in 30 min (II).

### 3.5. Polyacrylamide Gel Electrophoresis Under Denaturing Conditions

To assess the homogeneity of the oligonucleotides, analytical electrophoresis was carried out in 0.4 mm thick 20% polyacrylamide gel from acrylamide−*N*,*N*′-methylene-*bis*-acrylamide (30:1) at 50 V/cm voltage in 90 mM Tris-borate buffer, pH 8.3, containing 8 M urea and 2 mM Na_2_EDTA (all−Dia-M, Moscow, Russia). Oligonucleotides were loaded onto the gel as solutions containing 8 M urea, 0.05% Xylene Cyanol FF and 0.05% Bromophenol Blue (both Sigma-Aldrich Inc., St Louis, MO, USA). Bands were visualized by staining with a solution of Stains-All dye (500 mg/L) (Sigma-Aldrich Inc., St Louis, MO, USA) in formamide (Dia-M, Moscow, Russia), followed by rinsing with distilled water.

Oligonucleotides synthesized for UV melting studies were isolated by preparative polyacrylamide gel electrophoresis in 2–3 mm thick 20% gel under denaturing conditions (8 M urea) and desalted on a NAP-25 column with Sephadex G25 (GE Healthcare, Buckinghamshire, UK) in the form of sodium salts.

### 3.6. UV Melting Studies

RNA templates 5′-r(UGUUUGGCGC) and 5′-r(ATTTGAGCCTGGGAG) were kindly provided by Dr. Maria Meshchaninova. Thermal denaturation analysis of the duplexes of the modified oligonucleotides with complementary DNA or RNA was carried out in a quartz micro multi-cell cuvette with optical path length of 1 mm using a UV-1800i UV–Vis spectrophotometer (Shimadzu, Japan) with a Peltier thermoelectric unit. All the experiments were conducted in a buffer containing 10 mM sodium cacodylate, 5 mM MgCl_2_ and 100 mM NaCl, pH 7.4. The components were taken in stoichiometric ratios so that the total oligonucleotide duplex concentration in the buffer was 10 μM, with the exception of 20 nt oligonucleotides **Φ9** and **Φ10**, where it was 2.5 μM. The solutions were kept at 90 °C for 5 min, followed by gradual decrease in temperature from 90 °C to 15 °C to anneal the duplexes. Thereafter, the samples were heated from 15 °C to 90 °C at a rate of 0.2 °C min^−1^ to denature the duplex. Absorption spectra were recorded at 260 nm with a measurement step of 0.5 °C.

### 3.7. Cell Line

MDA MB 231 (human breast cancer cells) cells were cultivated in Iscove’s Modified Dulbecco’s Medium (IMDM) (Sigma-Aldrich Inc., St Louis, MO, USA) supplemented with 10% heat-inactivated fetal bovine serum (FBS) (GE Healthcare, Chicago, IL, USA) at 37 °C in a humidified atmosphere with 5% CO_2_ (standard conditions, SC) and passaged regularly to maintain exponential growth.

### 3.8. MTT Assay of Cell Viability

MDA MB 231 cells were seeded in 96-well plates at a density of 50 × 10^3^ or 1.5 × 10^4^ cells/well in complete IMDM supplemented by 10% FBS and incubated for 48 h under SC to adhere. Then, the medium was replaced with IMDM supplemented by 10% FBS containing a corresponding fluorinated oligonucleotide in concentrations from 0.1 to 50 µMm and incubated for 24 h under SC. Cytotoxicity of oligonucleotides was assessed by the MTT assay. Briefly, 12 mM MTT solution (Sigma-Aldrich, Darmstadt, Germany) was added to the cells, and the incubation was continued under SC for additional 4 h. Then, the MTT-containing medium was aspirated, and the formazan crystals formed in living cells were solubilized with 100 μL/well of SDS-HCl for additional 6 h. The absorbance of each well was read at the test and reference wavelengths (λ) of 570 and 620 nm, respectively, on a Multiscan RC plate reader (Thermo LabSystems, Helsinki, Finland). The percentage of living cells was calculated as follows:

Living cells (%) = (OD_570exp_ − OD_620exp_)/(OD_570cont_ − OD_620cont_) × 100,

where OD_570exp_ and OD_570cont_ correspond to optical density in experimental and control wells, respectively, at λ 570 nm, and OD_620exp_ and OD_620cont_ correspond to optical density in experimental and control wells, respectively, at λ 620 nm. All experimental points were run in triplicate for statistical analysis.

### 3.9. Confocal Microscopy

MDA MB 231 human breast cancer cells were seeded on coverslips (Marienfeld, Lauda-Königshofen, Germany) in 24-well plates at a density of 20 × 10^3^ cells/well in complete DMEM medium and incubated for 24 h under SC to adhere. Afterwards, MDA MB 231 cells were incubated in the corresponding complete media in the presence of 5 μM of the fluorinated oligonucleotide under SC for either 12 or 24 h. After incubation, coverslips with cells were washed twice with PBS, fixed with 3.7% formaldehyde in PBS buffer (15 min, 37 °C) and washed twice with PBS. The cells were stained with phalloidin-TRITC (ECM Biosciences, Versailles, KY, USA) according to the manufacturer’s protocols. After staining, the cells were washed twice with PBS and placed on slides in a drop of ProLong^TM^ Glass Antifade Mountant with NucBlue^TM^ (Thermo Fisher Scientific, Waltham, MA, USA). Mounted samples were allowed to cure on a flat, dry surface for 18–24 h in the dark at ambient temperature. Intracellular localization of fluorinated oligonucleotides was assessed by confocal fluorescent microscopy on an LSM710 (Zeiss, Oberkochen, Germany) using an αPlan-Apochromat 100×/1.46 Oil DIC M27 objective. Analysis of intracellular accumulation was performed using ZEN 2014 SP1 software (Zeiss, Oberkochen, Germany). Confocal microscopic analysis was performed in three channels (blue, green and red). Fluorescence in the blue channel corresponded to DAPI (nuclear staining), the green channel corresponded to fluorescence of oligonucleotides labeled with fluorescein (Flu), and the red channel corresponded to phalloidin-TRITC (cytoskeleton staining).

## 4. Conclusions

In this Communication, we described a convenient application of the Staudinger reaction to obtain new oligonucleotide derivatives containing polyfluoroalkyl groups at internucleotidic positions, which, due to the unique properties of fluorine atoms, can potentially improve the therapeutic utility of oligonucleotides. We have obtained oligonucleotides with one or two perfluoro-1-octanesulfonyl phosphoramidate groups (**Φ** series) or 2,2,2-trifluoroethanesulfonyl phosphoramidate groups (**Θ** series) up to complete substitution at all internucleotidic positions, as well as oligonucleotides with several fluorine-containing zwitter-ionic groups (**F** series). The incorporation of all these modifications did not require cumbersome synthetic steps, and the groups turned out to be stable under the conditions of automatic solid-phase DNA synthesis and standard ammoniacal deprotection. The stability of the duplexes formed by oligodeoxynucleotides with one or more polyfluorinated residues with complementary DNA or RNA were similar or only slightly less than the stability of the native DNA–DNA and DNA–RNA duplexes. Oligonucleotides with one or two perfluorooctyl groups were capable of gradual internalization by cells, although, seemingly, with less efficiency than the examples described in the literature for a different cell line [82].

To conclude, the oligonucleotides with polyfluorinated groups obtained have shown interesting properties that require more careful and comprehensive study on different cell lines to establish a reliable picture of the mechanism(s) of their cellular uptake, as well as to assess the influence of fluorinated groups on the biological activity of oligonucleotides. Further studies are planned to assess carefully both cytotoxicity and in vivo toxicity and the nanoparticle formation of polyfluoro oligonucleotides, as well as the application of the potential antisense and siRNA agents; the results will be published in due course.

## Figures and Tables

**Table 1:** Oligonucleotides with 2,2,2-trifluoroethanesulfonyl- and perfluoro-1-octanesulfonyl phosphoramidate groups obtained in this work.

Oligonucleotide Sequence, 5′-3′ *^a^*	Code	Oligonucleotide Sequence, 5′-3′	Code
DMTr-T^θ *b*^TTTTT ***^c^***	**Θ** *^b^***1**	GCGCCA^φ^AACA	**Φ4**
T^θ^TTTTT	**Θ** **2**	GCGCCA^φ^AAC^φ^A	**Φ5**
GCGCCAAAC^θ^A	**Θ** **3**	TG^φ^TTTGGCGC	**Φ6**
GCGCCA^θ^AACA	**Θ** **4**	TGTTT^φ^GGCGC	**Φ7**
GCGCCA^θ^AAC^θ^A	**Θ** **5**	TG^φ^TTT^φ^GGCGC	**Φ8**
G^θ^C^θ^G^θ^C^θ^C^θ^A^θ^A^θ^A^θ^C^θ^A	**Θ** **6**	TTTTTTTTTT^φ^TTTTTTTTTT	**Φ9**
T^θ^G^θ^T^θ^ T^θ^T^θ^G^θ^G^θ^C^θ^G^θ^C	**Θ** **7**	AAAAAAAAAA^φ^AAAAAAAAAA	**Φ10**
*G ^d^CGCC*AAA*C*^θ^A	**Θ** **8**	A^φ^GTCTCGACTTGCTACC	**Φ11**
*GCGCC*A^θ^AA*C*A	**Θ** **9**	A^φ^GTCTCGACTTGCTACC-Flu	**Φ12**
*GCGCC*A^θ^AA*C*^θ^A	**Θ** **10**	AGTCTCGACTTGCTAC^φ^C	**Φ13**
*G*^θ^*C*^θ^*G*^θ^*C*^θ^*C*^θ^A^θ^A^θ^A^θ^*C*^θ^A	**Θ** **11**	Flu-AGTCTCGACTTGCTAC^φ^C	**Φ14**
DMTr-T^φ *e*^TTTTT	**Φ** *^e^***1**	A^φ^GTCTCGACTTGCTAC^φ^C	**Φ15**
T^φ^TTTTT	**Φ2**	A^φ^GTCTCGACTTGCTAC^φ^C-Flu	**Φ16**
GCGCCAAAC^φ^A	**Φ3**	Flu-A^φ^GTCTCGACTTGCTAC^φ^C	**Φ17**

*^a^* The symbols (^θ^) and (^φ^) mark the positions of 2,2,2-trifluoroethanesulfonyl- and perfluoro-1-octanesulfonyl phosphoramidate groups, respectively. *^b^* Greek *theta*. *^c^* Straight capitals indicate oligodeoxynucleotides. *^d^* Italics designate 2′-*O*-methylribonucleotides. *^e^* Greek *phi*. Background color blue for oligonucleotides with 2,2,2-trifluoroethanesulfonyl phosphoramidate group; green for oligonucleotides with perfluoro-1-octanesulfonyl phosphoramidate groups. Flu—6-carboxamidofluorescein residue.

**Table 2:** Amide-linked polyfluorinated oligonucleotide conjugates with various amines obtained in this work.

Code	Oligonucleotide Sequence, 5′-3′ *^a^*	Amine R−NH_2_
**F1**	DMTr-T***^F^^i^***TTTTT ***^b^***	NH_3_/Pr*^i^*OH (***i***) R = H
**F2**	T***^F^^i^***TTTTT	
**F3**	DMTr-T***^F^^ii^***TTTTT	MeNH_2_/EtOH (***ii***) R = Me
**F4**	T***^F^^ii^***TTTTT	
**F5**	T***^F^^iii^***TTTTT	1,1-Dimethylethylenediamine (***iii***) R = −(CH_2_)_2_NMe_2_
**F6**	TTTTT***^F^^iii^***T	
**F7**	TTTT***^F^^iii^***T***^F^^iii^***T	
**F8**	T***^F^^iii^***TTTT***^F^^iii^***T	
**F9**	T***^F^*^iii^**T***^F^^iii^***TT***^F^^iii^***T***^F^^iii^***T	
**F10**	T***^F^^iii^***T***^F^^iii^***T***^F^^iii^***T***^F^^iii^***T***^F^^iii^***T	
**F11**	CTCCCAGGCTCAAA***^F^^iii^***T	
**F12**	CTC CCAGG***^F^^iii^***CTCAAAT	
**F13**	CTCCCAGGCTCAA***^F^^iii^***A***^F^^iii^***T	
**F14**	C***^F^^iii^***TCCCAGGCTCAAA***^F^^iii^***T	
**F15**	CTCCCAGG***^F^^iii^***CTCAAA***^F^^iii^***T	
**F16**	T***^F^^iv^***TTTTT	1,3-Diaminopropane (***iv***) R = −(CH_2_)_3_NH_2_
**F17**	TTTTT***^F^^iv^***T	
**F18**	TTTT***^F^^iv^***T***^F^^iv^***T	
**F19**	T***^F^^iv^***TTTT***^F^^iv^***T	
**F20**	T***^F^^iv^***T***^F^^iv^***TT***^F^^iv^***T***^F^^iv^***T	
**F21**	T***^F^^iv^***T***^F^^iv^***T***^F^^iv^***T***^F^^iv^***T***^F^^iv^***T	

*^a^* The symbol (***^F^***) marks the positions of fluorine-containing ester group, and the superscript Roman numerals (***^i^*^−*iv*^**) next to the letter refers to the corresponding conjugated amine. *^b^* All the oligonucleotides were oligodeoxyribonucleotides; prefix “d” was omitted throughout.

**Table 3:** Thermal stability of the complementary duplexes of modified polyfluoro oligonucleotides with DNA and RNA.

Code	Oligonucleotide Sequence, 5′-3′		*T*_m_, °C	Δ*T*_m_*,* °C		*T*_m_, °C		Δ*T*_m_*,* °C	
			DNA Template 5′-d(TGTTTGGCGC)			RNA Template 5′-r(UGUUUGGCGC)			
Control	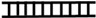	GCGCCAAACA	54.3 ± 0.2	−		49.1 ± 0.2		–	
**Θ3**	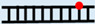	GCGCCAAAC^θ^A	54.6 ± 0.2	+0.3 ± 0.3		49.6 ± 0.2		+0.5 ± 0.3	
**Θ4**	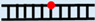	GCGCCA^θ^AACA	54.9 ± 0.1	+0.6 ± 0.2		48.9 ± 0.1		–0.2 ± 0.3	
**Θ5**	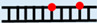	GCGCCA^θ^AAC^θ^A	53.9 ± 0.3	−0.4 ± 0.4		48.5 ± 0.1		–0.6 ± 0.3	
**Θ6**	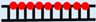	G^θ^C^θ^G^θ^C^θ^C^θ^A^θ^A^θ^A^θ^C^θ^A	50.8 ± 0.3	−3.6 ± 0.4		41.4 ± 0.2		–7.6 ± 0.3	
**Φ3**	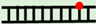	GCGCCAAAC^φ^A	54.5 ± 0.5	0		–		–	
**Φ4**	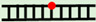	GCGCCA^φ^AACA	53.9 ± 0.5	0		–		–	
			DNA template 5′-d(ATTTGAGCCTGGGAG)			RNA template 5′-r(AUUUGAGCCUGGGAG)			
Control	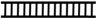	CTCCCAGGCTCAAAT	61.0 ± 0.4	−		65.7 ± 0.5		–	
**F11**	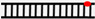	CTCCCAGGCTCAAA***^F^*^3^**T	59.6 ± 0.7	−1.4 ± 0.8		65.0 ± 0.9		–0.7 ± 1.0	
**F12**	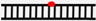	CTC CCAGG***^F^*^3^**CTCAAAT	59.4 ± 0.5	−1.6 ± 0.6		65.7 ± 0.6		–0.0 ± 0.8	
**F13**	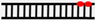	CTCCCAGGCTCAA***^F^*^3^**A***^F^*^3^**T	60.7 ± 0.1	−0.3 ± 0.4		65.9 ± 0.1		–0.2 ± 0.5	
**F14**	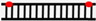	C***^F^*^3^**TCCCAGGCTCAAA***^F^*^3^**T	59.7 ± 0.6	−1.3 ± 0.7		65.7 ± 0.9		–0.0 ± 1.0	
**F15**	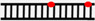	CTCCCAGG***^F^*^3^**CTCAAA***^F^*^3^**T	61.8 ± 0.3	+0.8 ± 0.5		64.7 ± 0.6		–1.0 ± 0.8	

				***T*** **_m_** **, °C**			**Δ*T*_m_*,* °C**		
Control	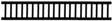	5′-d(TTTTTTTTTTTTTTTTTTTT)-3′ 3′-d(AAAAAAAAAAAAAAAAAAAA) -5′		50.7 ± 0.2			−		
**Φ9** Control	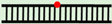	5′-d(TTTTTTTTTT^φ^TTTTTTTTTT)-3′ 3′-d(AAAAAAAAAA AAAAAAAAAA)-5′		48.4 ± 0.1			−2.4 ± 0.3		
**Φ9:Φ10**	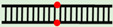	5′-d(TTTTTTTTTT^φ^TTTTTTTTTT)-3′ 3′-d(AAAAAAAAAA^φ^AAAAAAAAAA) -5′		44.6 ± 0.1			−6.2 ± 0.3		
**Φ3:Φ6**	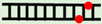	5′-d(GCGCCAAAC^φ^A)-3′ 3′-d(CGCGGTTT^φ^GT)-5′		51.7 ± 0.1			−2.6 ± 0.3		
**Φ4:Φ7**	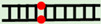	5′-d(GCGCCA^φ^AACA)-3′ 3′-d(CGCGGT^φ^TTGT)-5′		49.5 ± 0.5			−4.9 ± 0.3		
**Θ6:Θ7**	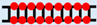	5′-d(G^θ^C^θ^G^θ^C^θ^C^θ^A^θ^A^θ^A^θ^C^θ^A)-3′ 3′-d(C^θ^G^θ^C^θ^G^θ^G^θ^T^θ^T^θ^T^θ^G^θ^T)-5′		47.7 ± 0.2			−6.7 ± 0.2		

Black ladder symbolizes a duplex with DNA or RNA; red dots signify the positions of the modifications. For oligonucleotide codes, see Table 1.

## Data Availability

All the data obtained are available in the main paper and in the respective Supplementary Materials.

## Supplementary Materials

The following supporting information can be downloaded at: https://www.mdpi.com/article/10.3390/ijms26010300/s1.
