# Supplementary material for: Synthesis of New Polyfluoro Oligonucleotides via Staudinger Reaction
Source: Int J Mol Sci. 2024 Dec 31;26(1):300. doi: 10.3390/ijms26010300 (PMC11719919; doi:10.3390/ijms26010300)
Supplement: Supplementary file 1 [file ijms-26-00300-s001.zip › ijms-3391814-supplementary.pdf]

Supplementary Material

Synthesis of new polyfluoro oligonucleotides *via* Staudinger reaction

Kristina Klabenkova[1,2], Alyona Zakhryamina[3], Ekaterina Burakova[1,2], Sergei Bizyaev[1,2], Alesya Fokina[1,2] and Dmitry Stetsenko[1,2*]

[1] Department of Physics, Novosibirsk State University, 2 Pirogov str., Novosibirsk 630090, Russia

[2] Institute of Cytology and Genetics, Siberian Branch of the Russian Academy of Sciences, 10 Lavrentiev Ave., Novosibirsk 630090, Russia

[3] Department of Natural Sciences, Novosibirsk State University, 2 Pirogov str., Novosibirsk 630090, Russia

* Correspondence: stetsenkoda@bionet.nsc.ru; Tel.: +7 (383) 363-49-63 ext. 8114.

General information

All reactions were carried out under argon atmosphere using anhydrous solvents unless otherwise stated. Standard *N*-protected deoxyribonucleoside 5′-DMTr-3′-β-cyanoethyl-*N*,*N*-diisopropyl phosphoramidites (Sigma-Aldrich Inc., St Louis, MO, USA) and 500Å CPG polymer supports (Glen Research Corp, Sterling, VA, USA) were used for oligonucleotide synthesis. Acetonitrile (UHPLC Supergradient, Panreac, Spain) for oligonucleotide synthesis was refluxed over CaH_2 under argon for 6 h, distilled and stored under argon over 3Å molecular sieves. All amines used for conjugation were from Sigma-Aldrich (Saint Louis, MO, USA). For HPLC, Supergradient UHPLC grade acetonitrile (Panreac, Madrid, Spain) and triethylammonium acetate (TEAA) buffer, pH 7.0 (Thermo Fisher Scientific, USA) were used. Stains-All, Xylene Cyanol FF, and Bromophenol Blue (BP) dyes together with sodium cacodylate were from Sigma-Aldrich (Saint Louis, MO, USA), sodium and lithium perchlorate from Acros Organics (Carlsbad, CA, USA). Formamide, acrylamide, *N*,*N′*-methylene-*bis*-acrylamide, urea, *tris*(hydroxymethyl)-aminomethane (Tris), boric acid, and disodium ethylenediaminetetraacetate were from Dia-M (Moscow, Russia). Conc. aq. ammonia solution and acetone (purest grade) were from SoyuzKhimProm (Novosibirsk, Russia). Reagents and solvents were purchased from their respective commercial suppliers, and used without further purification unless otherwise stated. Bi-distilled water was prepared in the laboratory.

For centrifugation of small volumes, a MiniSpin Plus microcentrifuge (Eppendorf, Hamburg, Germany) was used. Chemical reactions are carried out using a Thermomixer Compact thermoshaker (Eppendorf, Hamburg, Germany). The solutions were shaken using a BioVortex V1 vortex (Biosan, Riga, Latvia). Gel electrophoresis was carried out using an electrophoresis unit from Bio-Rad (Hercules, CA, USA). Small volumes of oligonucleotide solutions up to 1.5 mL were evaporated in a Concentrator Plus vacuum concentrator (Eppendorf, Hamburg, Germany). Oligonucleotide solutions, after purification, were lyophilized using a FreeZone freeze-drier (Labconco, Kansas City, MO, USA). Mass spectra were recorded using a ESI LC-MS/MS Agilent G6410A mass spectrometer (Agilent Technologies, USA). The optical densities of the solutions of oligonucleotide conjugates were measured using a NanoDrop 2000c (ThermoFisher Scientific, USA). Thermal denaturation curves were recorded on a UV-1900i UV-VIS spectrophotometer (Shimadzu, Japan) equipped with Peltier unit.

^{1}H NMR spectra were recorded in solution (20–40 mg/mL) on Bruker DRX-500, Avance 400, or Avance 300 spectrometers (500, 400, and 300 MHz, respectively). ^{13}C NMR spectra were acquired on Bruker DRX-500 or Avance 400 spectrometers (125 and 100 MHz, respectively). The residual solvent signals were used as internal standards for ^{1}H and ^{13}C NMR ($CDCl_3$: 7.24 ppm for ^{1}H, 76.9 ppm for ^{13}C; acetone-d_6: 2.05 ppm for ^{1}H, 29.84 ppm for ^{13}C). The assignment of signals in the ^{13}C NMR spectra was done based on calculations within the framework of the DFT ($CHCl_3$ as the solvent). Signal assignment was made using J modulated ^{13}C NMR spectra (proton-noise decoupling, the opposite phases for the signals of the atoms with the odd and even numbers of the attached protons, tuning to the constant *J* = 135 Hz) and 2D NMR spectra: 1) homonuclear ^{1}H◎^{1}H correlation, 2) heteronuclear ^{13}C◎^{1}H correlation at the direct spin-spin coupling constants (*J* = 135 Hz), 3) heteronuclear ^{13}C◎^{1}H correlation at the long range spin-spin coupling constants (*J* = 10 Hz), and 4) homonuclear J-resolved ^{1}H spectra. Carbon-proton spin-spin coupling constants were taken from proton-coupled ^{13}C NMR spectra. Sign of spin-spin couplings was not determined. Chemical shifts (◎ values) are expressed in ppm, coupling constants (*J*) are expressed in Hz,

and multiplicities are mentioned as follows: s (singlet), d (doublet), t (triplet), q (quartet), m (multiplet). Width at half-height ($W_{1/2}$) for broadened signals is given in Hz. Critical resolution parameters when registering 1D NMR spectra: spectral width 5500 Hz (11 ppm) for ^{1}H and 31.44 KHz (250 ppm) for ^{13}C, size of FID 16K for ^{1}H and 32K for ^{13}C, size of real spectrum 128K for both ^{1}H and ^{13}C, spectral resolution 0.042 Hz for ^{1}H and 0.24 Hz (0.002 ppm) for ^{13}C.

IR spectra were recorded on a Bruker Vector 22 spectrometer. Merck silica gel Kieselgel 60 (63–100 µ) was used for flash chromatography. Monitoring of the reaction progress was done by taking ^{1}H NMR spectra of the reaction mixture, and by TLC on Sorbfil plates (visualization by iodine vapors or UV irradiation).

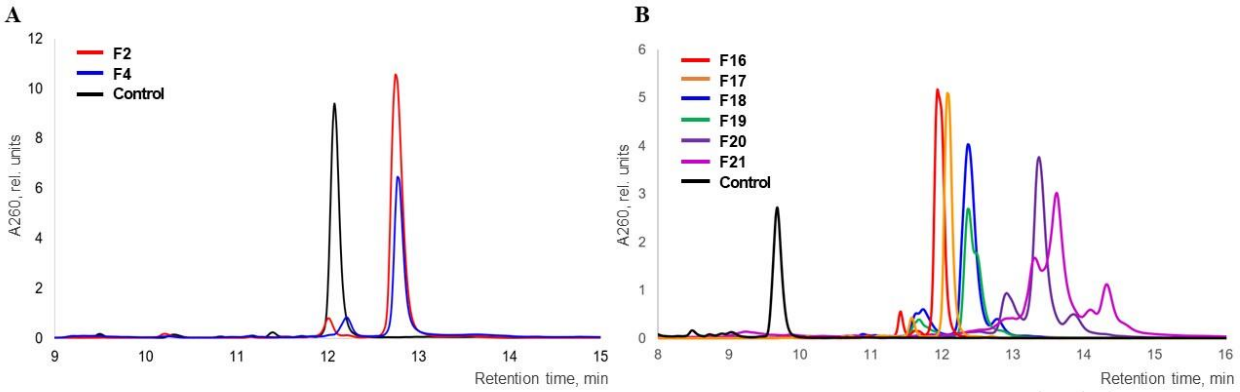

Figure S1. RP-HPLC elution profiles: (**A**) conjugates **F2**, **F4**, treated with solutions NH_3/*i*PrOH and CH_3NH_2/EtOH, respectively, compared to unmodified oligonucleotide 5′-d(TTTTTT)-3′, elution gradient (*i*); (**B**) conjugates **F16-F21** with 1,3-diaminopropane, compared to unmodified oligonucleotide 5′-d(TTTTTT)-3′, elution gradient (*ii*).

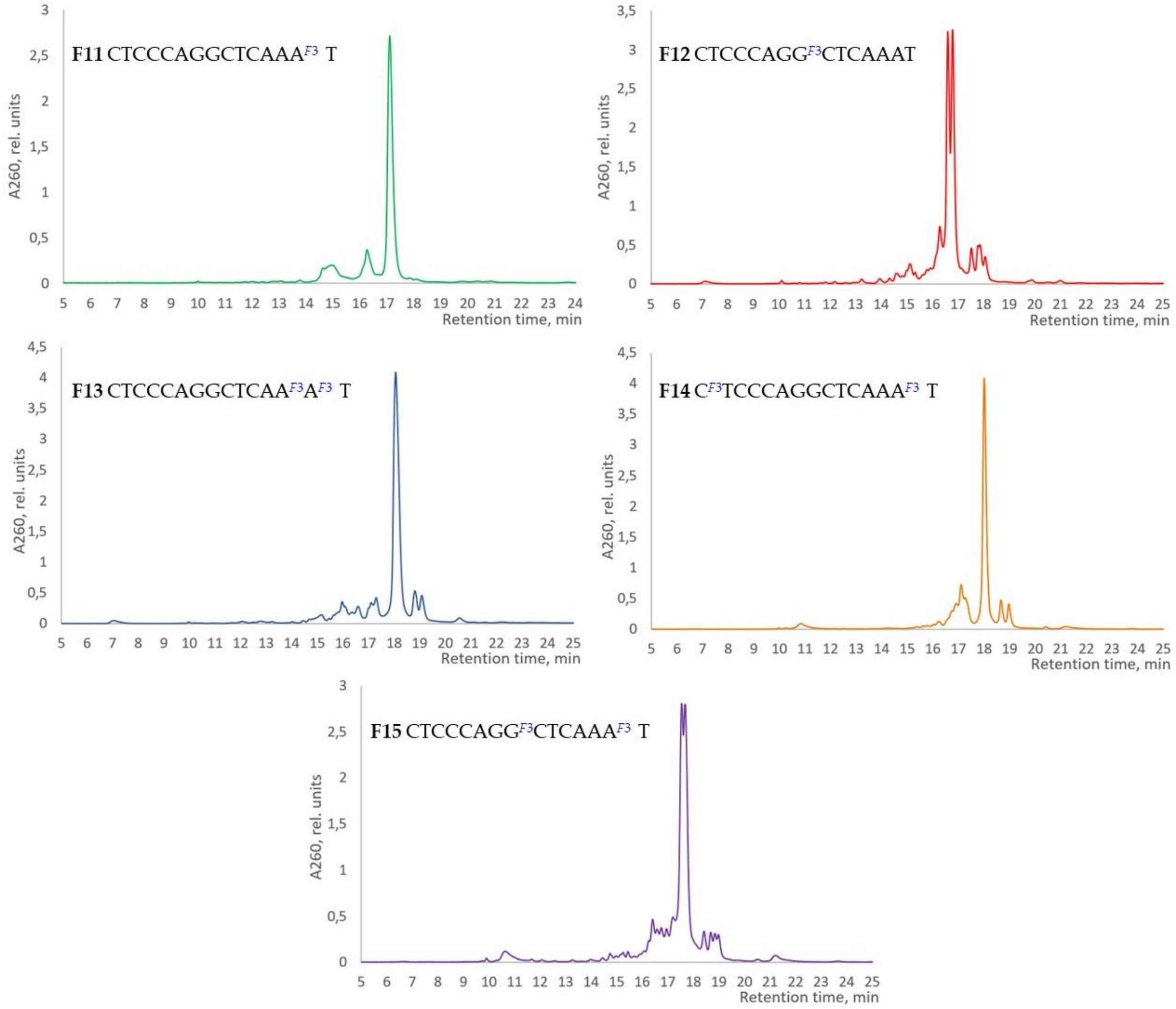

Figure S2. RP-HPLC profiles of crude oligonucleotides **F11-F15**.

Table S1. ESI LC-MS/MS of modified oligonucleotides

Oligonucleotide sequence, 5′-3′ [a]	Molecular mass, Da		Code
	Calc. [M-2H]$^{-}$	Exp. [M-2H]$^{-}$	
DMTr-T$^{\varphi}$TTTTT [b]	2546.66	2546.66	**Φ1**
T$^{\varphi}$TTTTT	2244.32	2244.37	**Φ2**
DMTr-T$^{\theta}$TTTTT	2210.66	2210.65	**Θ1**
T$^{\theta}$TTTTT	1908.3	1908.3	**Θ2**
GCGCCAAAC$^{\varphi}$A	3487.07	3488.05	**Φ3**
GCGCCA$^{\varphi}$AACA	3487.07	3487.75	**Φ4**
GCGCCA$^{\varphi}$AAC$^{\varphi}$A	3968.2	3969.94	**Φ5**
TG$^{\varphi}$TTTGGCGC	3531.07	3531.55	**Φ6**
TGTTT$^{\varphi}$GGCGC	3531.07	3531.55	**Φ7**
TG$^{\varphi}$TTT$^{\varphi}$GGCGC	4012.2	4012.44	**Φ8**
TTTTTTTTTT$^{\varphi}$TTTTTTTTTT	6502.95	6502.31	**Φ9**
AAAAAAAAAA$^{\varphi}$AAAAAAAAAA	6683.15	6683.25	**Φ10**
GCGCCAAAC$^{\theta}$A	3151.10	3151.13	**Θ3**
GCGCCA$^{\theta}$AACA	3151.10	3150.85	**Θ4**
GCGCCA$^{\theta}$AAC$^{\theta}$A	3296.21	3296.20	**Θ5**
G$^{\theta}$C$^{\theta}$G$^{\theta}$C$^{\theta}$C$^{\theta}$A$^{\theta}$A$^{\theta}$A$^{\theta}$C$^{\theta}$A	4311.93	4311.89	**Θ6**
T$^{\theta}$G$^{\theta}$T$^{\theta}$T$^{\theta}$T$^{\theta}$G$^{\theta}$G$^{\theta}$C$^{\theta}$G$^{\theta}$C	4355.93	4346.86	**Θ7**

[a] All the oligonucleotides were oligodeoxynucleotides, prefix ‘d’ was omitted throughout. [b] The symbols ($^{\theta}$) and ($^{\varphi}$) mark the positions of 2,2,2-trifluoroethanesulfonyl phosphoramidate and perfluoro-1-octanesulfonyl phosphoramidate groups, respectively. DMTr – 4,4′-dimethoxytrityl.

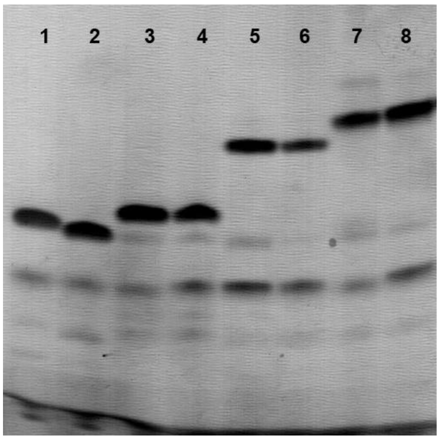

Figure S3. Electrophoretic comparison of the mobility of oligonucleotides conjugated with different amines; lanes: (*1*) – unmodified 5′-d(TTTTT), (*2*) – unmodified 5′-d(TTTTTT), (*3*) – sample **F2** 5′-d(T^{F1}TTTTT), (*4*) – sample **F4** 5′-d(T^{F2}TTTTT), (*5*) – sample **F5** 5′-d(T^{F3}TTTTT), (*6*) – sample **F6** 5′-d(TTTTT^{F3}T), (*7*) – sample **F16** 5′-d(T^{F4}TTTTT) and (*8*) – sample **F17** 5′-d(TTTTT^{F4}T) (see Table 2 in the main paper).

Syntheses of perfluoro-1-octanesulfonyl azide (2a), 2,2,2-trifluoroethanesulfonyl azide (2b), and 2,2-difluoro-3-azidosulfonylacetate (2c).

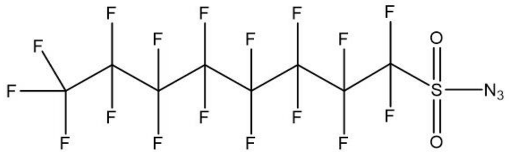

Perfluoro-1-octanesulfonyl azide (**2a**). Perfluoro-1-octanesulfonyl fluoride (1 eq, 275 µL, 502 mg, 1 mmol) was added to a suspension of sodium azide NaN_3 (1.2 equiv, 78 mg, 1.2 mmol) in 4 ml acetone under argon atmosphere. The suspension was shaken vigorously for 5 minutes then left on a shaker at 1,400 rpm for 72 h at 25°C. The sodium fluoride suspension was precipitated by centrifugation for 15 min at 3,500 rpm, and

the solution of perfluoro-1-octanesulfonyl azide was carefully transferred into a 15 ml plastic tube. Afterwards, to a solution of perfluoro-1-octanesulfonyl azide in acetone was added acetonitrile in a 1:1 ratio and stored resulted solution (~0.1 M) in the dark under argon atmosphere.

IR: N_3-group = 2120 cm^{-1}; RSO_2 = 1370 cm^{-1}. ^{19}F NMR (400 MHz, Acetone-d_6, δ, ppm): 34,8-35,1 (m, CF_3); 38,3 - 41,3 (m, α – CF_2); 53,2 – 53,8 (m, β – CF_2); 79,4 – 81,2 (m, 3x CF_2); 88,8 – 91,2 (m, ζ – CF_2); 207,2-207,6 (m, θ – CF_2).

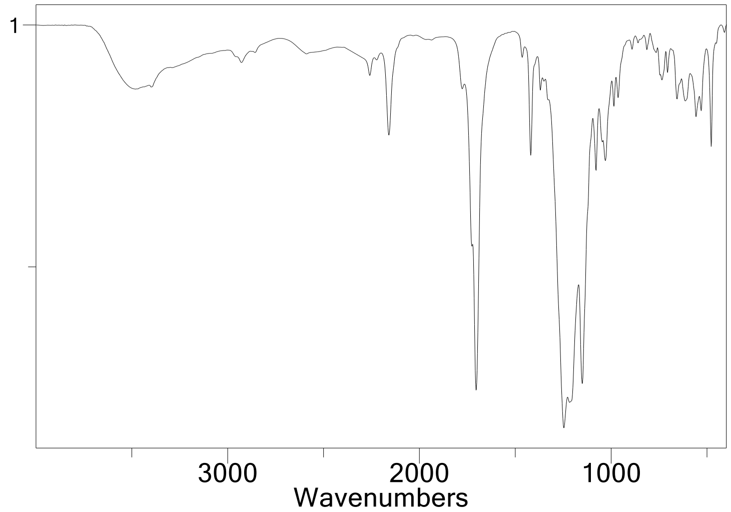

Figure S4. IR spectrum of perfluoro-1-octanesulfonylazide (**2a**).

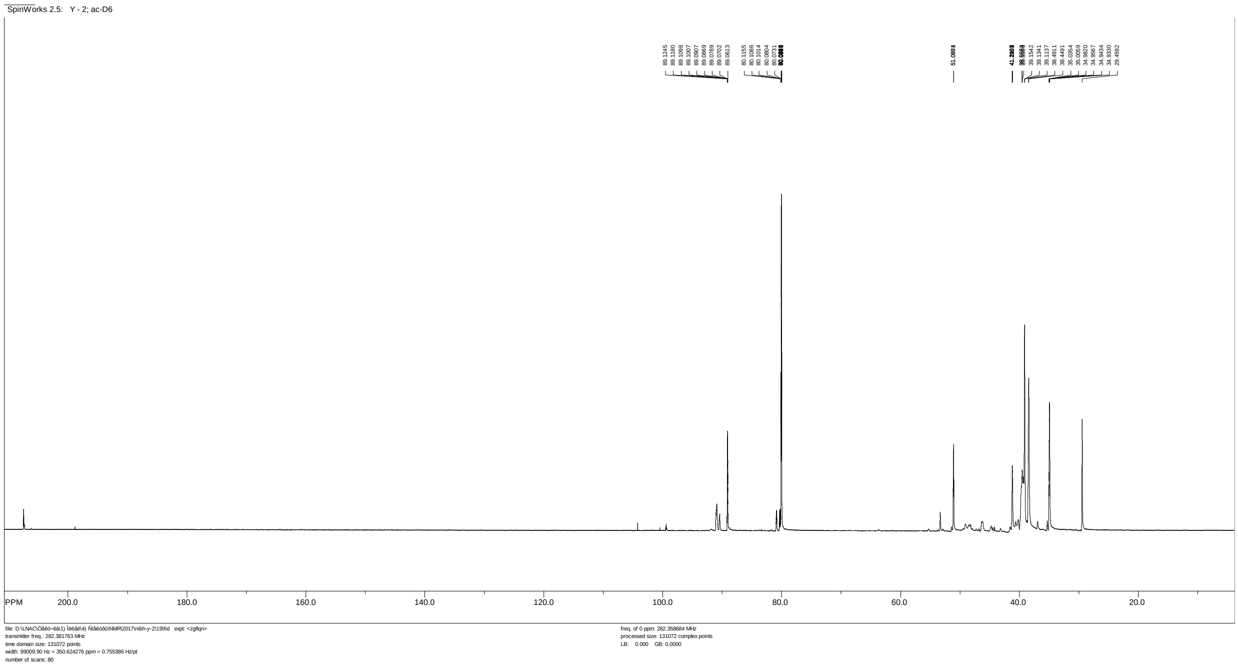

Figure S5. ^{13}F spectrum of perfluoro-1-octanesulfonyl azide (**2a**).

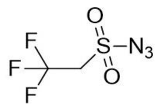

2,2,2-Trifluoroethanesulfonyl azide (**2b**). 2,2,2-Trifluoroethanesulfonyl (tresyl) chloride (1 eq., 199 μL, 328 mg, 1.5 mmol) was added to a suspension of sodium azide NaN_3 (1.2 equiv, 140 mg, 1.8 mmol) in 12 ml of

dry acetonitrile under argon atmosphere. The suspension was shaken vigorously for 5 minutes then left on a shaker at 1400 rpm for 12 h at 25°C. Afterwards, the sodium chloride suspension was precipitated by centrifugation for 15 min at 3,500 rpm, the solution of 2,2,2-trifluoroethanesulfonyl azide (~0.15 M) was carefully transferred into a 15 ml plastic tube, and stored in the dark under argon atmosphere.

IR: N_3-group = 2170 cm^{-1}; RSO_2 = 1350 cm^{-1}. ^{19}F NMR (400 MHz, CD_3CN, δ, ppm): 101.73 (t, J = 9.1 Hz, 3F); ^1H NMR (400 MHz, CD_3CN, δ, ppm): 4.22 (q, J = 8.8 Hz, 2H); ^{13}C NMR (500 MHz, CD_3CN, δ, ppm): 122.1 (q, CF_3, $J_{C\text{-}F}$ = 266 Hz); 57.13 (q, CH_2, J_2 = 32.6 Hz).

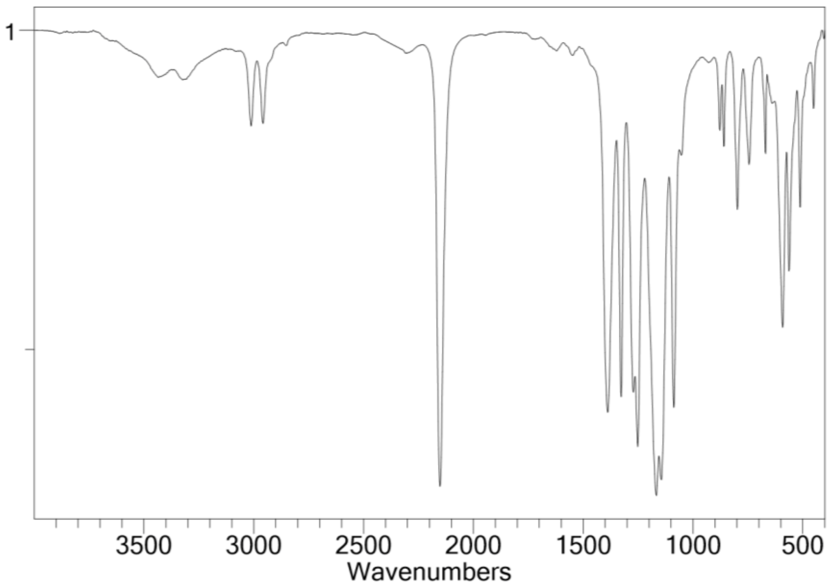

Figure S6. IR spectrum of of 2,2,2-trifluoroethanesulfonylazide (**2b**).

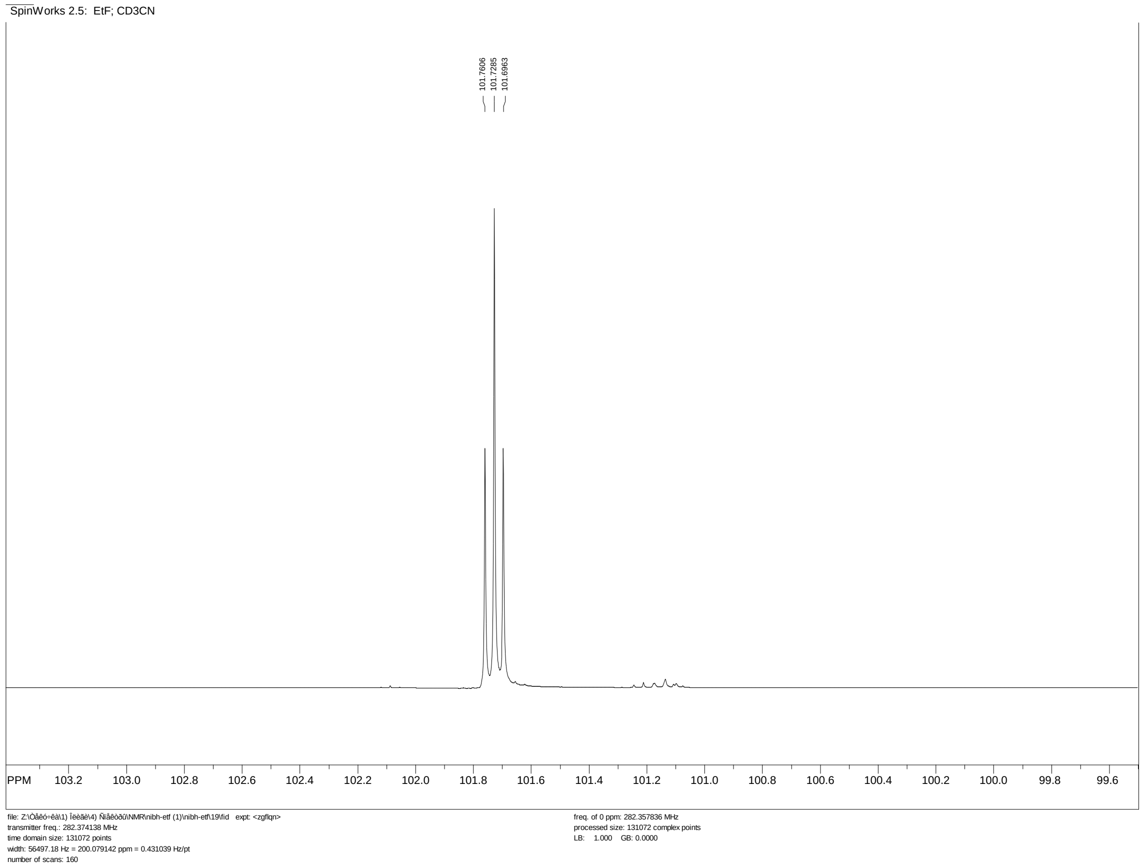

Figure S7. ^{19}F NMR spectrum of 2,2,2-trifluoroethanesulfonyl azide (**2b**).

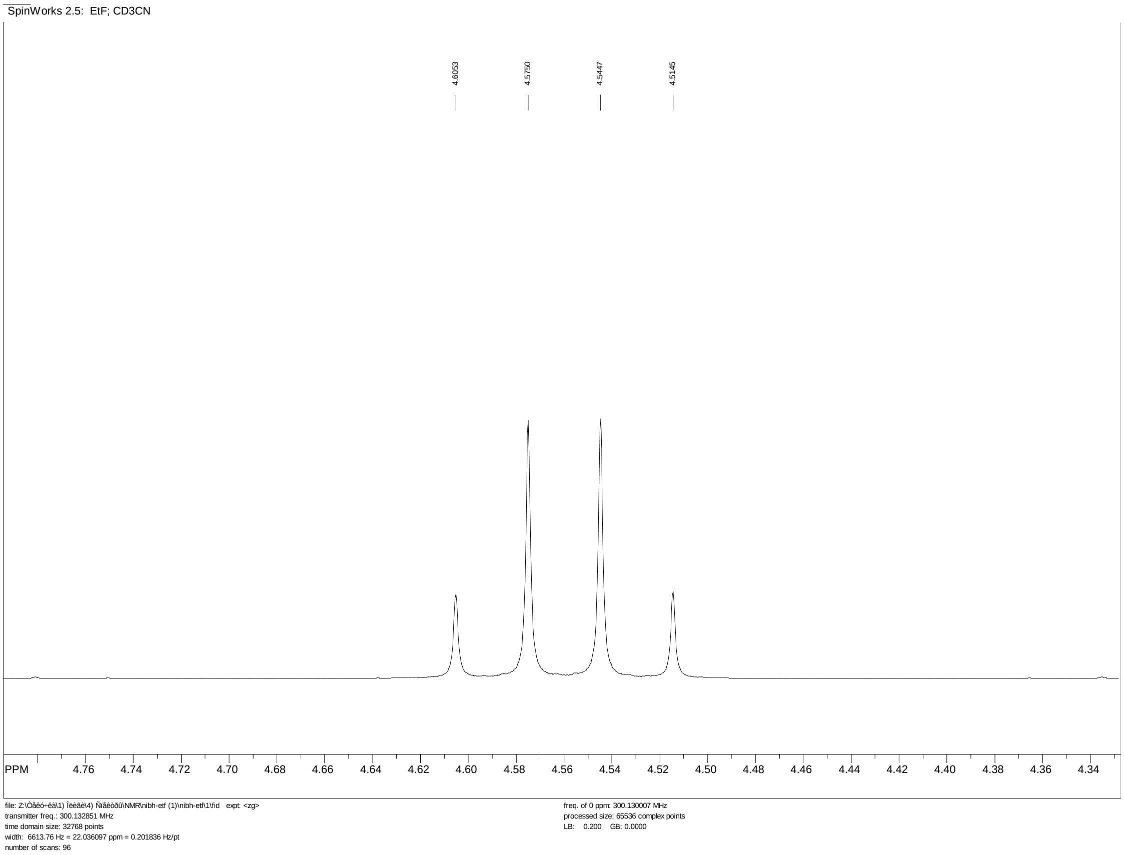

Figure S8. ^{1}H NMR spectrum of 2,2,2-trifluoroethanesulfonyl azide (**2b**).

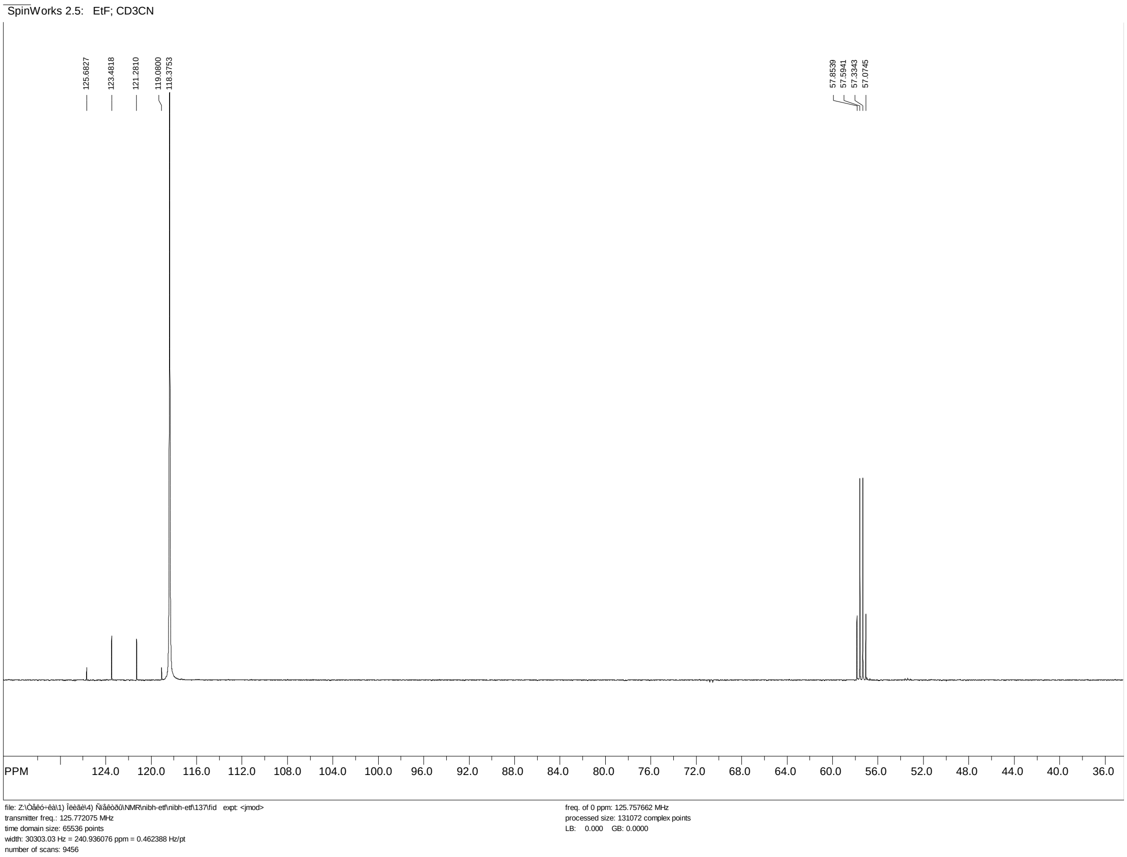

Figure S9. ^{13}C NMR spectrum of of 2,2,2-trifluoroethanesulfonyl azide (**2b**).

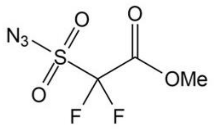

2,2-Difluoro-2-(azidosulfonyl)acetate (**2c**). A modified procedure of Barrow and Moses was used (Barrow, A.S.; Moses, J.E. Synthesis of Sulfonyl Azides via Lewis Base Activation of Sulfonyl Fluorides and Trimethylsilyl Azide. *SynLett*. **2016**, *27*, 1840-1843, doi: 10.1055/s-0035-1561626). Methyl-2,2-difluoro-2-(fluorosulfonyl)acetate (1 eq., 306 μL, 461 mg, 2.4 mmol) was added to a suspension of sodium azide NaN_3 (1.5 equiv, 243 mg, 3.6 mmol) in 11 ml of dry acetonitrile under argon atmosphere. In additional tube, 4-dimethylaminopyridine (0.1 eq., 29 mg, 0.24 mmol) was dissolved in 695 μL of dry acetonitrile and the resulting solution was added to the reaction mixture. The suspension was shaken vigorously for 10 minutes then left on a shaker at 1,400 rpm for 24 h at 25°C. The sodium fluoride suspension was precipitated by centrifugation for 15 min at 3,500 rpm and the solution of 2,2-difluoro-2-(azidosulfonyl)acetate was carefully transferred into a pure 15 ml plastic tube. The solution was kept in the dark over 3Å molecular sieves under argon atmosphere for two days. Prior to the transferring to the synthesizer bottle, 600 μL of 2,6-luitidine (5 vol. %) was added to the solution (~0.25 M). ^{1}H NMR (400 MHz; $CDCl_3$; δ, ppm): 4.03 (s, 3H). ^{19}F NMR (282 MHz; $CDCl_3$; δ, ppm): –107.40 (2F).

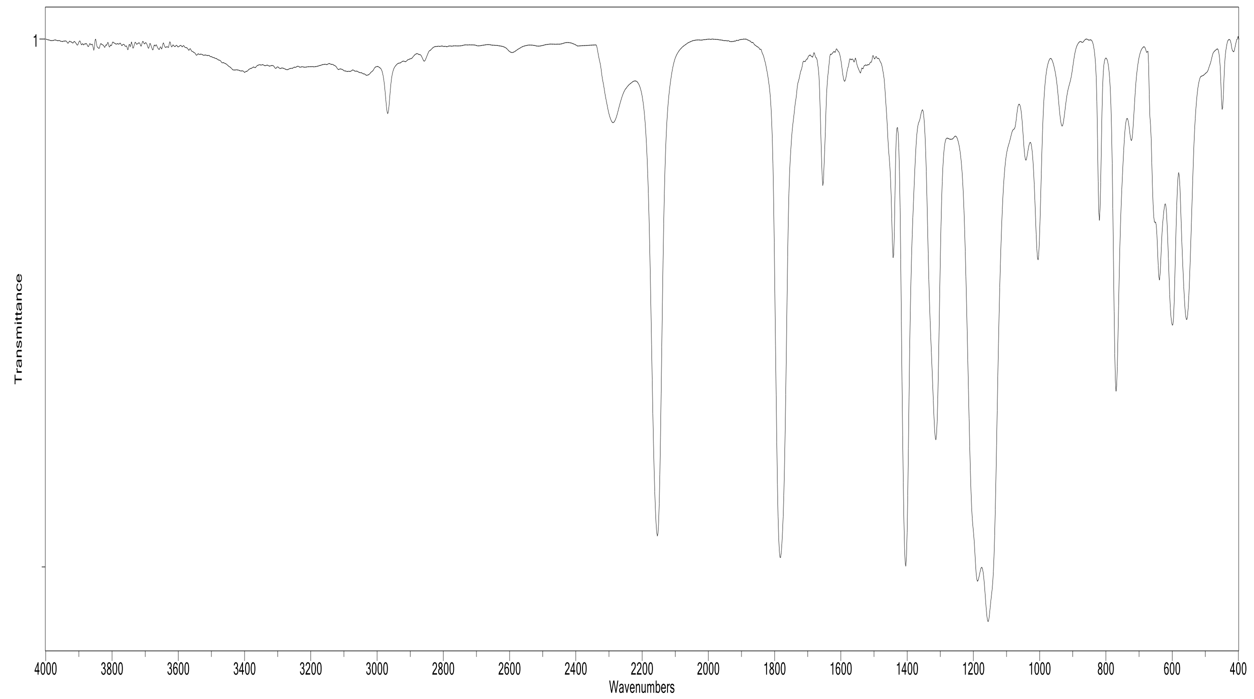

Figure S10. IR spectrum of 2,2-difluoro-2-(azidosulfonyl)acetate (**2c**).

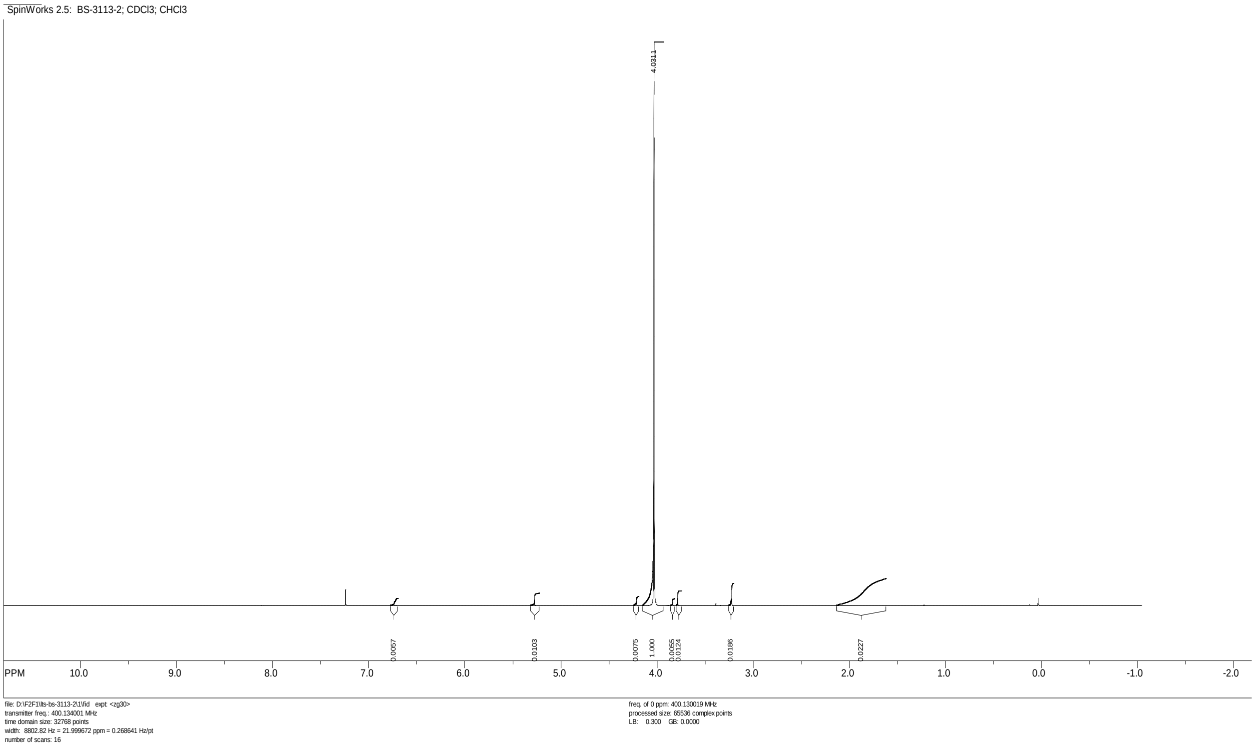

Figure S11. ^{1}H NMR spectrum of 2,2-difluoro-2-(azidosulfonyl)acetate (**2c**).

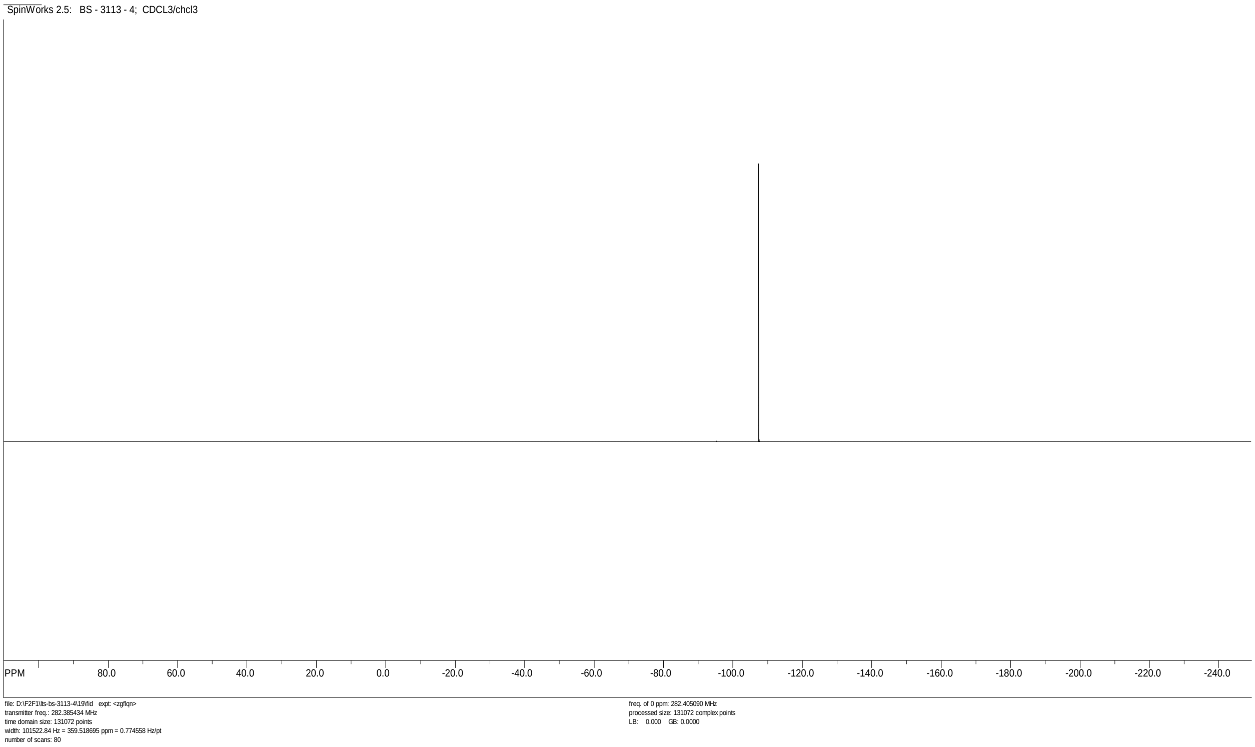

Figure S12. ^{19}F NMR spectrum of 2,2-difluoro-2-(azidosulfonyl)acetate (**2c**).
